# Preparation, Spectroscopic Elucidation, Antioxidant Potential, Enzyme Inhibition, and Molecular Docking Studies of Novel Schiff Base‐Derived Thiosemicarbazones

**DOI:** 10.1002/cbdv.71882

**Published:** 2026-10-30

**Authors:** Hasan Yakan, Halit Muglu, Semiha Yenigun, Tevfik Ozen

**Affiliations:** ^1^ Department of Chemistry Education Faculty of Education Ondokuz Mayıs University Samsun Türkiye; ^2^ Department of Chemistry Faculty of Sciences Kastamonu University Kastamonu Türkiye; ^3^ Department of Chemistry Faculty of Sciences Ondokuz Mayıs University Samsun Türkiye

**Keywords:** DPPH^•^ scavenging activity, enzyme inhibitors, molecular docking, structure elucidation, thiosemicarbazones

## Abstract

New thiosemicarbazones bearing Schiff base (**1–7**) were prepared from 4‐(dimethylamino)benzaldehyde and thiosemicarbazides. The reaction of various isothiocyanates with hydrazine monohydrate resulted in the thiosemicarbazides. The structural frameworks of the newly developed derivatives were confirmed by elemental analysis and conventional spectroscopic methods, namely, FT‐IR, ^1^H, and ^13^C NMR. The *in vitro* antioxidant activity of the synthesized series was evaluated using the DPPH^•^ (1,1‐diphenyl‐2‐picrylhydrazyl) scavenging method. The results indicated that the inhibitory concentrations (IC_50_) of the compounds ranged from 8.50 ± 0.14 to 31.44 ± 1.39 µg/mL, with compound **6** exhibiting the most potent antioxidant activity. Additionally, the inhibitory activities of urease and xanthine oxidase (XO) were assessed, and the interactions of the compounds with these enzymes were also analyzed. Molecular docking was performed to assess the interactions between each molecule and the active sites of urease and XO.

## Graphical abstract

The synthesis of new thiosemicarbazones bearing Schiff base was performed. The structures of the compounds were characterized through several spectroscopic methods. The antioxidant potential of these compounds was evaluated through DPPH^•^ *in vitro* assays. In addition, their inhibitory activities against urease and xanthine oxidase (XO) were investigated. Furthermore, molecular docking were performed to explore potential binding interactions between the synthesized compounds and the target enzymes.

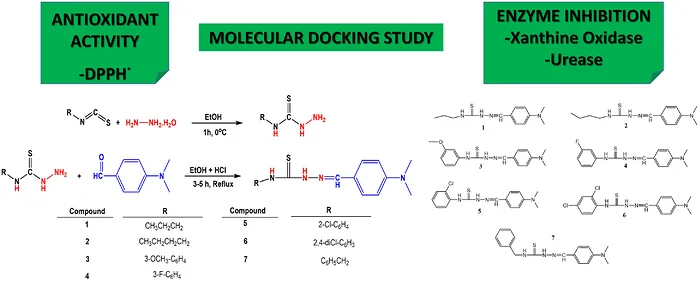

## 1 Introduction

Thiosemicarbazones are a noteworthy group of organosulfur compounds in the realm of synthetic organic chemistry, defined by the distinctive ─NH─C(═S)NH─N═ functional unit. Owing to their structural diversity, they are widely used as key intermediates in the synthesis of various biologically active compounds. Over the years, these molecules have attracted considerable attention due to their broad spectrum of pharmacological and biological properties, such as anticancer [1], antitubercular [2], anticonvulsant [3], urease inhibitor [4, 5], antiviral [6], antibacterial [7], antimicrobial [8, 9], and antioxidant [10, 11, 12, 13] properties. Recently, there has been a growing emphasis on Schiff base‐integrated thiosemicarbazones, given their notable efficacy demonstrated in various biological evaluations. These compounds have exhibited significant biological activities, particularly antibacterial and antifungal [14, 15], antioxidant [12, 16], antimicrobial [17, 18], enzyme inhibitory properties [19, 20, 21, 22, 23], ameliorative effect [24], anti‐HIV [25], anticancer [26], and anti‐inflammatory [27] activities.

Antioxidants are increasingly recognized as a promising approach for treating various lifestyle‐related diseases [28, 29]. Oxidative stress plays a pivotal role in the pathogenesis of various health conditions, including cancer, diabetes, cardiovascular diseases, and the accelerated aging process. Furthermore, the generation of reactive oxygen species (ROS) is closely linked to genomic instability, often resulting in significant DNA mutations and damage [30, 31]. Many antioxidant compounds and enzymes play a protective role against these harmful effects. Consequently, numerous researchers have focused on developing and synthesizing compounds with antioxidant properties [10, 32, 33, 34].

Ureases are widely distributed in nature across prokaryotes and eukaryotes, including bacteria, fungi, algae, plants, and invertebrates [35]. Elevated ammonia levels in the stomach create a favorable environment for the survival of *Helicobacter pylori*. Bacterial ureases are associated with a range of diseases, including peptic ulcers, kidney stones, hepatic encephalopathy, pyelonephritis, and hepatic coma [36, 37, 38]. One of the most promising and effective therapeutic strategies for managing gastric complications caused by hyperactive urease is the use of synthetic small molecules to inhibit urease activity. This approach helps to regulate urease function and alleviate related health issues.

Elevated uric acid levels are often linked to increased xanthine oxidase (XO) activity and oxidative stress, both of which are associated with various vascular diseases [39]. One consequence of excessive XO activity is gout, a common form of inflammatory arthritis [40]. Acute pain and joint swelling are primarily driven by the localized deposition of uric acid crystals, a condition that stems from elevated systemic levels of this metabolic byproduct. To manage hyperuricemia and gout, treatment strategies generally focus on either enhancing uric acid elimination or reducing its production [41]. XO inhibitors play a vital role in this approach. By inhibiting XO activity, these drugs help lower uric acid levels in the bloodstream and associated oxidative stress, thereby offering therapeutic benefits for patients with gout and related conditions [42].

Therefore, the present study aimed to design and synthesize seven novel thiosemicarbazone derivatives incorporating Schiff base moieties and to investigate their structural and biological properties comprehensively. FT‐IR and ^1^H and ^13^C NMR spectroscopy were used to characterize the synthesized compounds structurally. Their antioxidant potential was evaluated using the DPPH free radical scavenging assay, and their inhibitory activities against urease and XO were determined by *in vitro* enzyme inhibition assays. To gain insight into these interactions, molecular docking was conducted to examine how each compound binds to the active sites of urease and XO.

## 2 Results and Discussion

### 2.1 Physical Properties

Tables 1 and 2 present the comprehensive data regarding the elemental analyses and physicochemical properties of the series.

**TABLE 1:** Physicochemical properties of the synthesized compounds.

Comp.	Structure name	M.P., °C	Yield, %	Color
**1**	*N*‐propyl‐2‐(4‐(dimethylamino)benzylidene)hydrazine‐1‐carbothioamide	167–168	63	Yellow
**2**	*N*‐butyl‐2‐(4‐(dimethylamino)benzylidene)hydrazine‐1‐carbothioamide	179–180	81	Orange
**3**	*N*‐(3‐methoxyphenyl)‐2‐(4‐(dimethylamino)benzylidene)hydrazine‐1‐carbothioamide	187–188	84	Orange
**4**	*N*‐(3‐fluorophenyl)‐2‐(4‐(dimethylamino)benzylidene)hydrazine‐1‐carbothioamide	169–170	78	Red
**5**	*N*‐(2‐chlorophenyl)‐2‐(4‐(dimethylamino)benzylidene)hydrazine‐1‐carbothioamide	179–180	67	White
**6**	*N*‐(2,4‐dichlorophenyl)‐2‐(4‐(dimethylamino)benzylidene)hydrazine‐1‐carbothioamide	198–199	79	Yellow
**7**	*N*‐benzyl‐2‐(4‐(dimethylamino)benzylidene)hydrazine‐1‐carbothioamide	147–148	75	Orange

**TABLE 2:** The data derived from the elemental analysis of the compounds.

Comp.	Molecular mass (g/mol)	Molecular formula	Calculated			Experimental		
			C%	H%	N%	(C)%	(H)%	(N)%
**1**	264	C_13_H_20_N_4_S	59.06	7.62	21.19	59.01	7.60	21.22
**2**	278	C_14_H_22_N_4_S	60.40	7.96	20.12	60.45	7.93	20.09
**3**	328	C_17_H_20_N_4_OS	62.17	6.14	17.06	62.11	6.16	17.04
**4**	316	C_16_H_17_FN_4_S	60.74	5.42	17.71	60.79	5.43	17.70
**5**	332.5	C_16_H_17_ClN_4_S	57.73	5.15	16.83	57.76	5.14	16.85
**6**	367	C_16_H_16_Cl_2_N_4_S	52.32	4.39	15.25	52.30	4.39	15.26
**7**	312	C_17_H_20_N_4_S	65.35	6.45	17.93	65.42	6.43	17.95

### 2.2 Vibrational Frequencies

The disappearance of the characteristic symmetric and asymmetric ─NH_2_ stretching bands in the 3400–3300 cm^−1^ region confirmed the consumption of the starting materials. Instead, the emergence of new absorption bands between 1607 and 1550 cm^−1^, assigned to the ─C═N stretching of the imine moiety, successfully verified the formation of the Schiff base structure. For all compounds (**1–7**), ─NH stretching vibrations associated with the thiosemicarbazone scaffold were identified within the 3309–3127 cm^−1^ range. Key signals for ─C═S and ─C─N (S═C─N and R─C─N) vibrations were observed at 1409–1355, 1300–1214, and 1276–1155 cm^−1^, respectively (for the spectra of all compounds, see Figures S1–S7). Specific substitutions were also evident: ─C─O at 1047 cm^−1^ for compound **3**, ─C─F at 1146 cm^−1^ for compound **4**, and ─C─Cl vibrations at 1060 and 1053 cm^−1^ for compounds **5** and **6**. These IR data, summarized in Table 3, align well with the literature reported for structurally related molecules [43, 44, 45].

**TABLE 3:** Experimental IR vibration frequencies (cm^−1^) of the compounds.

Comp.	*ν*_NH_	*ν*_C─H_ (Aromatic)	*ν*_C═H_ (Aliphatic)	*ν*_C═N_	*ν*_C═S_	*ν*_S═C─N,_ *ν*_ArC─N_	Subs.
**1**	3329, 3183	3006–2964	2922–2802	1594	1409	1245,1215	—
**2**	3309, 3180	3005–2954	2924–2798	1596	1364	1234,1207	—
**3**	3281, 3127	2989–2957	2919–2825	1589	1363	1272,1155	C─O: 1047
**4**	3279, 3149	3049–2989	2902–2820	1550	1367	1273,1232	C─F: 1146
**5**	3297, 3142	3059–2970	2885–2801	1603	1362	1214,1175	C─Cl:1060
**6**	3294, 3142	3024–2987	2890–2802	1607	1355	1254,1208	C─Cl: 1053
**7**	3303, 3189	3058–3002	2900–2802	1590	1363	1300,1276	—

**Abbreviation**: Subs, substituent.

### 2.3 ^1^H NMR Spectral Interpretations

The ^1^H NMR spectra of compounds (**1**–**7**) were recorded in DMSO‐*d_6_*, and detailed chemical shift values are compiled in Table 4. The thiosemicarbazone ‐NH proton (H5) appeared as a characteristic singlet within the 11.87–11.17 ppm region across all derivatives. Similarly, the imine methine proton (─CH═N, H4) was detected as a sharp singlet resonating between 8.07 and 7.93 ppm (for the spectra of all compounds, see Figures S8–S14). The dimethylamino group's methyl protons (─N(CH_3_)_2_, H1) were consistently observed as singlets in the 3.00–2.96 ppm range. In the aromatic region, H2 and H3 protons were detected between 7.72 and 6.69 ppm. Specifically, the H2 protons manifested as doublets at 7.72–7.58 ppm due to coupling with H3, while the H3 protons appeared as doublets at 6.75–6.69 ppm. For compounds **3–6**, the amino signal (─NH, H6) was resonated as a singlet at 10.02–9.94 ppm. For compounds **1**, **2**, and **7**, the amino signal (─NH, H6) was resonated as a triplet at 8.90–8.29 ppm due to the methylene group (─CH_2_). For compounds **3–7**, the aromatic proton (H7–H11) signals were detected in the ranges of 7.94–6.99 ppm. For compounds **3** and **7**, the methoxy (─OCH_3_) and the methylene group (N─CH_2_) proton signals were detected as a singlet at 3.77 and a doublet at 4.85–4.83 ppm, respectively. For compounds **1** and **2**, the proton signals of the propyl and butyl groups resonated in the ranges of 3.53–0.86 and 3.57–0.90 ppm, respectively. The observed signals correspond closely to previously published data for similar structures [17, 46, 47]. The signals for DMSO‐*d_6_* and water in DMSO (HOD, H_2_O) were observed at approximately 2.50 ppm (quintet) and 3.30 ppm, respectively, with the latter varying with solvent and concentration [48].

**TABLE 4:** Experimental ^1^H NMR data of the compounds (*δ*, ppm, in DMSO‐*d_6_*).

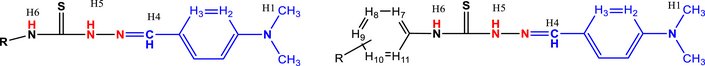							
Comp.	H1, CH_3_	H2	H3	H4, CH═N	H5, NH	H6, NH	*R*/H7–H11
**1**	2.97 (s, 6H)	7.60–7.58 (d, *J* = 8.8 Hz, 2H)	6.73–6.70 (d, *J* = 8.8 Hz, 2H)	7.93 (s, 1H)	11.18 (s, 1H)	8.34–8.31 (t, *J* = 5.6 Hz, 1H)	3.53–3.48 (dd, *J* = 13.8, 6.7 Hz, 2H), 1.64–1.55 (dq, *J* = 14.7, 7.3 Hz, 2H), 0.90–0.86 (t, *J* = 7.4 Hz, 3H)
**2**	2.97 (s, 6H)	7.60–7.58 (d, *J* = 8.8 Hz, 2H)	6.73–6.70 (d, *J* = 8.8 Hz, 2H)	7.93 (s, 1H)	11.17 (s, 1H)	8.32–8.29 (t, *J* = 5.7 Hz, 1H)	3.57–3.52 (dd, *J* = 13.8, 6.7 Hz, 2H), 1.60–1.53 (dt, *J* = 14.9, 7.6 Hz, 2H), 1.36–1.27 (dq, *J* = 14.8, 7.3 Hz, 2H), 0.93–0.90 (t, *J* = 7.4 Hz, 3H)
**3**	2.98 (s, 6H)	7.71–7.68 (d, *J* = 8.8 Hz, 2H)	6.74–6.72 (d, *J* = 9.5, 2H)	8.05 (s, 1H)	11.62 (s, 1H)	9.88 (s, 1H)	7.34 (s, 1H), 7.28–7.21 (m, 2H), 6.78–6.75 (dd, *J* = 6.4 Hz, 2.4 Hz, 1H), OCH_3_: 3.77 (s, 3H)
**4**	3.00 (s, 6H)	7.72–7.69 (d, *J* = 8.7 Hz, 2H)	6.74–6.72 (d, *J* = 8.9 Hz, 2H)	8.07 (s, 1H)	11.74 (s, 1H)	10.02 (s, 1H)	7.67 (s, 1H), 7.51‐7.49 (d, *J* = 7.7 Hz, 1H), 7.41–7.36 (dd, *J* = 15.6, 7.6 Hz, 1H), 7.04–6.99 (td, *J* = 8.5, 2.5 Hz, 1H)
**5**	2.98 (s, 6H)	7.66–7.64 (d, *J* = 8.9 Hz, 2H)	6.75–6.73 (d, *J* = 8.9 Hz, 2H)	8.05 (s, 1H)	11.81 (s, 1H)	9.95 (s, 1H)	7.94–7.91 (dd, *J* = 8.0, 1.4 Hz, 1H), 7.56–7.54 (dd, *J* = 8.0, 1.4 Hz, 1H), 7.40–7.36 (td, *J* = 7.8, 1.4 Hz, 1H), 7.30–7.26 (td, *J* = 7.8, 1.6 Hz, 1H)
**6**	2.98 (s, 6H)	7.67–7.64 (d, *J* = 8.8 Hz, 2H)	6.75–6.73 (d, *J* = 8.9 Hz, 2H)	8.05 (s, 1H)	11.87 (s, 1H)	9.94 (s, 1H)	7.89–7.87 (d, *J* = 8.6 Hz, 1H), 7.73–7.72 (d, *J* = 2.4 Hz, 1H), 7.48–7.45 (dd, *J* = 8.6, 2.4 Hz, 1H)
**7**	2.96 (s, 6H)	7.62–7.60 (d, *J* = 8.9 Hz, 2H)	6.71–6.69 (d, *J* = 8.9 Hz, 2H)	7.97 (s, 1H)	11.36 (s, 1H)	8.90–8.87 (t, *J* = 6.2 Hz, 1H)	7.37–7.22 (m, 5H), N─CH2: 4.85–4.83 (d, J = 6.2 Hz, 2H)

*Note*: s (singlet), d (doublet), dd (doublet of doublet), t (triplet), td (triplet of doublet), and m (multiplet).

### 2.4 ^13^C NMR Spectral Interpretations

The ^13^C NMR spectra for the synthesized series (**1**–**7**) were obtained in DMSO‐d_6_, and the corresponding chemical shifts are compiled in Table 5. The thiosemicarbazone scaffold was characterized by distinctive ─C═S signals resonated between 177.32 and 175.02 ppm. Additionally, the imine carbon (─CH═N) peaks were identified within the 144.93–143.34 ppm range. The methyl carbon (─CH_3_) resonances were consistently recorded between 40.25 and 40.20 ppm. In the aromatic region, the aldehyde‐derived carbon signals (C2‐C5) were localized at 152.12–151.79 ppm. Notably, the C2 resonance was observed further downfield compared to the other phenyl carbons, a shift attributed to the electronegative influence of the adjacent nitrogen atom (for the spectra of all compounds, see Figures S15–S21). For compound **1**, the carbon atom signals of the propyl group were resonated at 45.53 (N─CH_2_), 22.79 (─CH_2_─), and 11.74 (─CH_3_) ppm, respectively. For compound **1**, the carbon atom signals of the butyl group were resonated at 43.55 (N─CH_2_), 31.69 (─CH_2_─), 20.08 (─CH_2_─), and 14.31 (─CH_3_) ppm, respectively. For compounds **3–7**, the aromatic carbon atom (C8–C13) signals were observed in the ranges of 163.17–110.89 ppm. Some carbon atom signals were shifted downfield (high values of *δ*) relative to the signal of phenyl carbon (128.5 ppm) due to the presence of methoxy (─OCH_3_), nitrogen, ─F, ─Cl, atoms/groups. Furthermore, in compound **4**, the C atoms (for C8–C13) were also split into doublets due to interactions with the F nucleus. The methoxy (─OCH_3_) carbon signal of compound **3** was observed at 55.60 ppm. For compound **7**, the carbon signal of the methylene group (─CH_2_) was detected at 46.96 ppm. The obtained results align well with values reported in earlier studies of similar compounds [17, 46, 47].

**TABLE 5:** The experimental ^13^C NMR data of chemical shifts (*δ*, ppm) for the compounds.

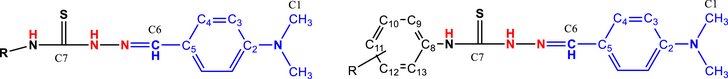														
Comp.	C1	C2	C3	C4	C5	C6	C7	C8	C9	C10	C11	C12	C13	*R*
**1**	40.25	151.80	112.14	129.02	121.96	143.36	176.74	—	—	—	—	—	—	45.53, 22.79, 11.74
**2**	40.25	151.79	112.14	129.02	121.97	143.34	176.69	—	—	—	—	—	—	43.55, 31.69, 20.08, 14.31
**3**	40.25	152.01	111.25	129.15	121.60	144.52	175.06	140.79	110.89	159.45	112.12	129.53	117.78	55.60
**4**	40.20	152.08	112.31	129.61	121.46	144.93	175.02	141.52, 141.41	112.09, 111.91	163.17, 160.77	111.70, 111.53	129.93, 129.83	121.30, 121.28	—
**5**	40.22	152.10	112.21	129.69	121.44	144.64	175.63	136.90	129.35	128.85	127.53	127.51	129.72	—
**6**	40.22	152.12	112.18	129.41	121.37	144.87	175.70	136.29	131.18	129.14	130.39	127.65	130.97	—
**7**	40.25	151.85	112.13	129.13	121.91	143.78	177.32	140.18	127.73	129.49	126.81	128.61	127.15	46.96

### 2.5 DPPH^•^ Scavenging Activity

The DPPH^•^ scavenging assay was employed to evaluate the antioxidant potential of the synthesized derivatives. Compounds with lower IC_50_ values exhibited stronger DPPH^•^ scavenging activity, indicating higher antioxidant potential. Evaluation of the DPPH^•^ scavenging activities revealed that all synthesized compounds, except for compound **5**, exhibited higher antioxidant activity than the reference standard, Trolox (Table 6). The free radical scavenging activities of the compounds decreased in the following order: **6 **> **1 **> **2 **> **7 **> **4 **> **3 **> trolox > **5**. Among the synthesized compounds, compound **6** (8.50 ± 0.14 µg/mL) displayed the strongest antioxidant activity, as evidenced by its lowest IC_50_ value.

**TABLE 6:** The antioxidant activity results of the obtained compounds.

Comp.	DPPH^•^ scavenging activity IC_50_ (µg/mL)	*R* ^2^
**1**	11.81 ± 0.50	0.974
**2**	12.79 ± 0.95	0.949
**3**	20.05 ± 1.26	0.973
**4**	17.45 ± 0.58	0.964
**5**	31.44 ± 1.39	0.929
**6**	**8.50 ± 0.14**	0.966
**7**	16.62 ± 1.15	0.959
**Trolox**	25.90 ± 0.42	0.910

*Note*: Values are expressed as mean (*n* = 3).

IC_50_ : Concentration (µg/mL) indicating 50% DPPH^•^ scavenging.

The IC_50_ values corresponding to the synthesized compounds were evaluated from 8.50 ± 0.14 to 31.44 ± 1.39 µg/mL. The electron‐donating or withdrawing substituents attached to the main scaffolds play a significant role in determining antioxidant activity, as previously highlighted [33, 43, 49]. According to the reaction DPPH^•^ + R‐XH → DPPH‐H + RX^•^ (where X═N, ─NH─C(═S)NH─N═, thiosemicarbazone moiety), compounds with weaker X─H bonds exhibit greater antioxidant activity because they can donate hydrogen atoms more readily. The strength of these bonds, particularly the ─NH bond in the thiosemicarbazone moiety and the ─OH bonds on the aldehydic phenyl ring, depends on the electron density surrounding the nitrogen and oxygen atoms. Consequently, the antioxidant activity of a compound is mainly determined by two factors: (1) its hydrogen atom‐donating ability and (2) the stability of the radical species formed after hydrogen donation [13, 33, 49].

Among the synthesized series, the 2,4‐dichloro substituted derivative compound **6** demonstrated a remarkable enhancement in total antioxidant capacity. This superior performance can be attributed to the presence of electron‐withdrawing chloro groups, which enhance the molecule's radical‐scavenging efficiency relative to other analogs. This behavior may be associated with the combined electronic and steric effects of the substituents, although the specific contributions of these factors were not directly investigated in the present study. As a result, hydrogen atom donation becomes more favorable, thereby enhancing antioxidant activity. For the remaining compounds, antioxidant activity was mainly governed by the stability of the radical species formed after hydrogen atom donation. As the stability of the resulting radical increased due to the electron‐donating substituents, the antioxidant activity followed the order: **1** (CH_3_CH_2_CH_2_) > **2** (CH_3_CH_2_CH_2_CH_2_) > **7** (C_6_H_5_CH_2_) > **4** (3‐F) > **3** (3‐OCH_3_) > **5** (2‐Cl), respectively. This trend may be attributed to the combined influence of the substituents' electron‐donating ability and steric properties. The alkyl groups in compounds **1** and **2** donate electron density through the positive inductive (+I) effect, which facilitates hydrogen atom donation and contributes to the stabilization of the resulting radical. Although the butyl group in compound **2** has a slightly stronger electron‐donating inductive effect, its larger size may introduce additional steric hindrance, slightly reducing the efficiency of hydrogen atom transfer. In contrast, the benzyl substituent in compound **7** is less effective in increasing electron density at the hydrogen‐donating site because the aromatic ring modifies the electronic effect of the methylene group, resulting in comparatively lower antioxidant activity. Among the substituted phenyl derivatives, the fluorine‐containing compound (**4**) showed higher activity than the methoxy (**3**) and chloro (**5**) analogs. This behavior may arise from the combined influence of the substituent's inductive and resonance effects, which alter the electron density around the hydrogen‐donating site, as well as differences in radical stabilization. The *ortho*‐chloro substituent in compound **5** may also introduce steric hindrance, reducing hydrogen atom transfer efficiency and resulting in the lowest antioxidant activity. Kiran et al. have previously shown that incorporating halogens into molecular structures can improve their antioxidant effectiveness [45]. Following the most potent derivative **6**, the compounds featuring propyl, butyl, and benzyl substitutions (**1**, **2**, and **7**, respectively) exhibited remarkable antioxidant efficiency. Notably, these three derivatives surpassed the radical scavenging capacity of the reference standard, Trolox. The *meta*‐fluoro‐ and *meta*‐methoxy‐substituted compounds **4** and **3** had higher activity than standard Trolox. The *ortho*‐chloro‐substituted compound **5** has the lowest antioxidant activity among the tested molecules and the standard.

### 2.6 Urease Inhibition Activity

While compounds **6**, **1**, and **2** showed higher urease inhibition than thiourea, the standard, compounds **4** and **7** showed inhibition levels close to thiourea. Other compounds were noted to show lower urease inhibition than thiourea (Table 7). The synthesized derivatives were ranked in descending order of their inhibitory potency as follows: **6 > 1 > 2 >** thiourea** > 4 > 7 > 3 > 5**.

**TABLE 7:** The urease and XO inhibitory results of the compounds.

Comp.	Urease inhibition activity (IC_50_, µg/mL)	XO inhibition activity (IC_50_, µg/mL)
**1**	8.74 ± 0.12	17.03 ± 0.61
**2**	9.61 ± 0.43	17.16 ± 0.82
**3**	13.65 ± 0.75	**5.53 ± 0.22**
**4**	11.56 ± 0.57	6.97 ± 0.20
**5**	20.55 ± 0.07	9.39 ± 0.10
**6**	**8.67 ± 0.14**	15.29 ± 0.44
**7**	11.78 ± 0.19	13.10 ± 0.60
Thiourea	10.67 ± 0.03	—
Allopurinol	—	11.94 ± 0.25

*Note*: Values are expressed as mean (*n* = 3).

IC_50_ : Concentration (µg/mL) indicating 50 % enzyme inhibition.

Compound **6**, which contains 2,4‐dichloro substitution, showed a marked increase in urease inhibition compared to the other compounds, likely due to the electron‐withdrawing nature of its substituents. Compounds **1** and **2** bearing propyl and butyl groups exhibited strong inhibitory activity, ranking just below compound **6** and surpassing the standard thiourea.

### 2.7 XO Inhibition Activity

In XO inhibition, the highest inhibitory activity was observed in compound **3**, followed by compounds **4** and **5**. Other compounds were determined to have lower XO inhibition than standard allopurinol. (Table 7). The compounds exhibited inhibitory activity in the following order: **3 **> **4 **> **5 **> Allopurinol > **7 **> **6 **> **1 **> **2**. The *meta*‐fluoro, *meta*‐methoxy, and *ortho*‐chloro‐substituted compounds **3‐5** had higher XO inhibition than standard allopurinol. These groups have electron‐withdrawing properties. The benzyl/propyl/butyl/ substituted compounds **7, 1,** and **2** showed a low inhibition due to the likely electron‐donating nature of its substituents, respectively.

### 2.8 Molecular Docking Study

Molecular docking was performed to investigate the possible binding modes of compounds **1–7** within the catalytic sites of urease and XO (Figures 1 and 2). The docking results were interpreted as computational estimates of ligand–enzyme interaction favorability rather than as direct measurements of experimental docking scores. The docking scores obtained for urease and XO are presented in Table 8, while the corresponding ligand–residue interaction profiles are provided in Tables 9 and 10.

**FIGURE 1:**
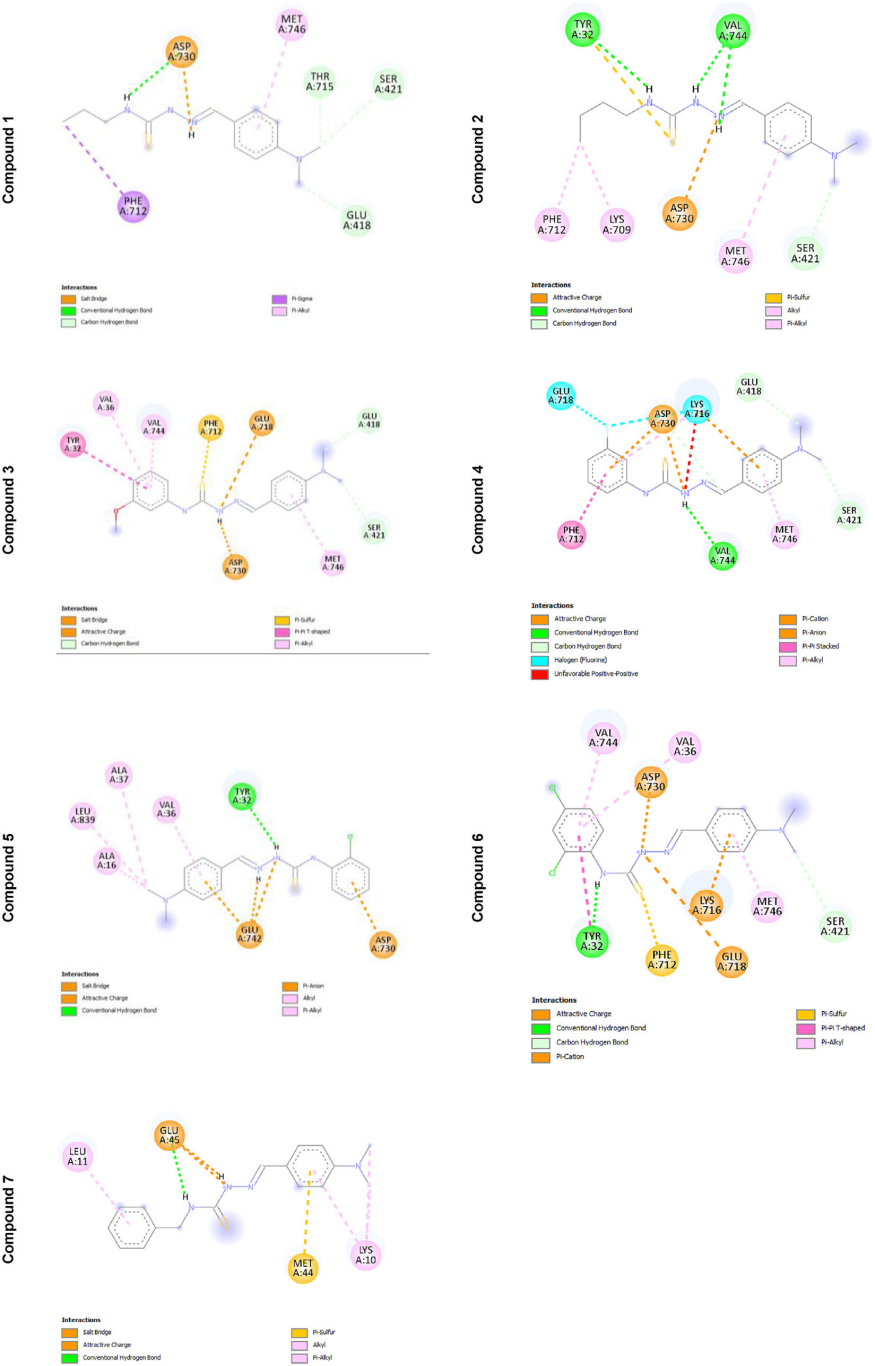
The 2D images of the interactions of compounds with urease.

**FIGURE 2:**
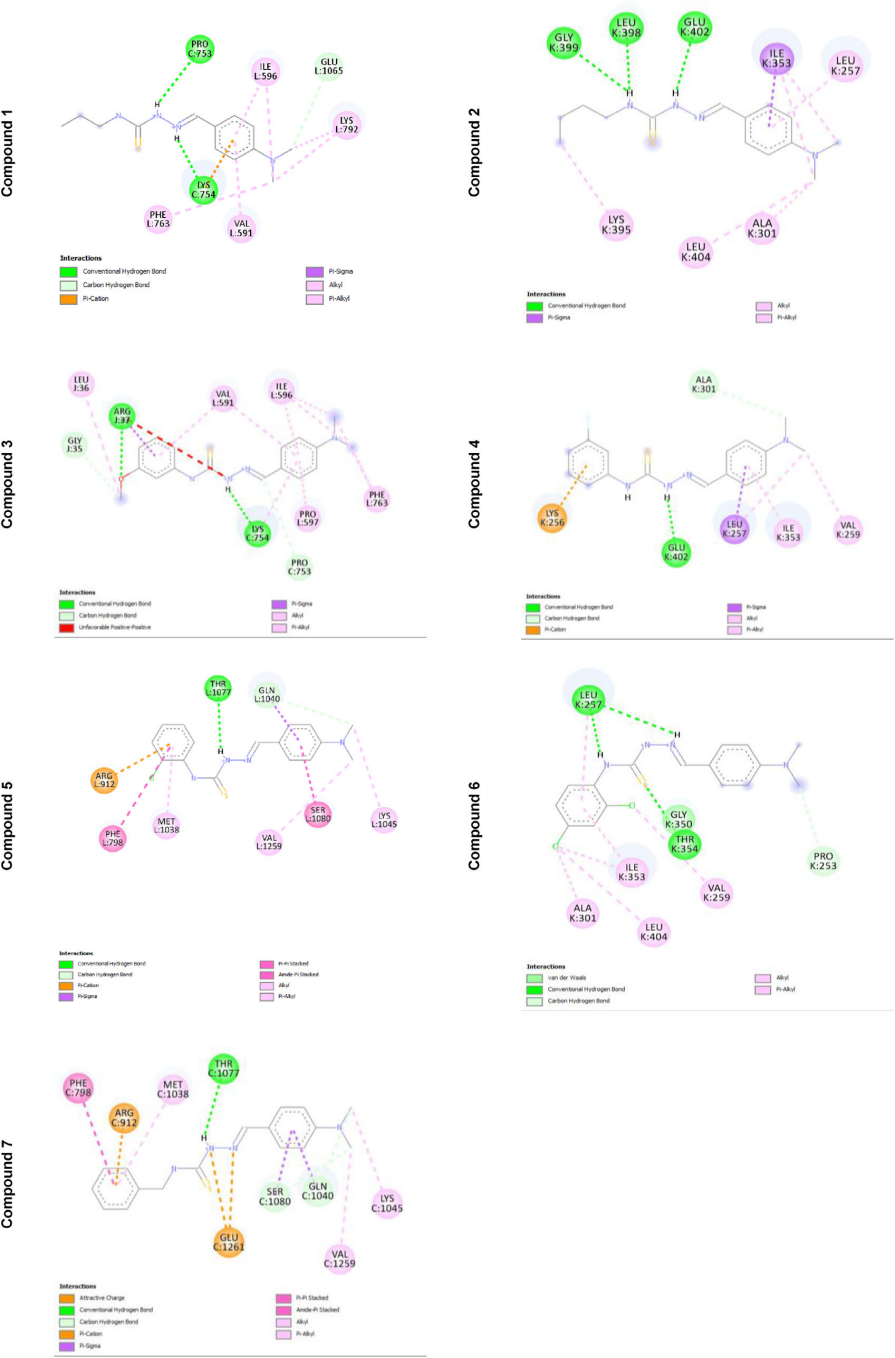
The 2D images of the interactions of compounds with XO.

**TABLE 8:** The docking scores of compounds with urease and XO.

Comp.	Urease	XO
	Docking scores, kcal/mol	Docking scores, kcal/mol
**1**	**−7.50**	−6.90
**2**	−7.20	−6.60
**3**	−7.20	−8.20
**4**	−5.60	**−8.30**
**5**	−5.80	−7.60
**6**	−7.30	−7.50
**7**	−6.20	−8.10
Thiourea	−3.20	—
Allopurinol	—	−5.80

**TABLE 9:** Urease‐compounds **1–7** interaction categories, species, and molecular docking distance.

Compound number	Interacting amino acids	Interaction distance (Å)	Interaction types
**1**	ASP730	2.11296	HB; electrostatic (Salt bridge)
	ASP730	2.05391	HB (Conventional)
	SER421	3.55325	HB (Carbon)
	THR715	3.65825	HB (Carbon)
	GLU418	3.44941	HB (Carbon)
	PHE712	3.97375	HYD (Pi–Sigma)
	LYS716	4.84015	HYD (Alkyl)
	LYS716	3.7885	HYD (Pi–Alkyl)
	MET746	5.18448	HYD (Pi–Alkyl)
**2**	ASP730	4.46172	Electrostatic (Attractive charge)
	VAL744	2.92556	HB (Conventional)
	VAL744	2.40197	HB (Carbon)
	TYR32	3.06572	HB (Carbon)
	SER421	3.60602	HB (Carbon)
	LYS716	4.74931	Electrostatic (Pi–Cation)
	TYR32	5.23064	Other (Pi–Sulfur)
	LYS709	4.7642	HYD (Alkyl)
	PHE712	3.94015	HYD (Pi–Alkyl)
	LYS716	4.41875	HYD (Pi–Alkyl)
	MET746	4.83961	HYD (Pi–Alkyl)
**3**	ASP730	2.51367	HB; electrostatic (Salt bridge; attractive charge)
	GLU718	5.21923	Electrostatic (Attractive charge)
	GLU418	3.40557	HB (Carbon)
	SER421	3.55182	HB (Carbon)
	PHE712	3.81551	Other (Pi–Sulfur)
	TYR32	5.40441	HYD (Pi–Pi T‐shaped)
	VAL36	5.29023	HYD
	VAL744	4.54653	HYD (Pi–Alkyl)
	LYS716	3.87533	HYD (Pi–Alkyl)
	MET746	4.89724	HYD (Pi–Alkyl)
**4**	ASP730	4.49131	Electrostatic (Attractive charge)
	VAL744	2.44843	HB (Conventional)
	ASP730	3.3066	HB (Carbon)
	GLU418	3.65934	HB (Carbon)
	SER421	3.48827	HB (Carbon)
	LYS716	2.7042	Halogen (Fluorine)
	GLU718	3.40213	Halogen (Fluorine)
	LYS716	4.89785	Electrostatic (Pi–Cation)
	ASP730	4.04715	Electrostatic (Pi–Anion)
	PHE712	3.92249	HYD (Pi–Pi Stacked)
	LYS716	5.40772	HYD (Pi–Alkyl)
	LYS716	4.45346	HYD (Pi–Alkyl)
	MET746	4.7131	HYD (Pi–Alkyl)
**5**	GLU742	2.56403	HB; electrostatic (Salt bridge; attractive charge)
	GLU742	2.54119	HB; electrostatic (Salt bridge; attractive charge)
	GLU742	3.17959	Electrostatic (Attractive charge)
	TYR32	2.28405	HB (Conventional)
	ASP730	3.93486	Electrostatic (Pi–Anion)
	GLU742	4.13521	Electrostatic (Pi–Anion)
	ALA16	3.58826	HYD (Alkyl)
	ALA37	4.37352	HYD (Alkyl)
	LEU839	4.15701	HYD (Alkyl)
	VAL36	5.03804	HYD (Pi–Alkyl)
**6**	GLU718	5.2177	Electrostatic (Attractive charge)
	ASP730	2.94862	Electrostatic (Attractive charge)
	TYR32	2.4193	HB (Conventional)
	SER421	3.47236	HB (Carbon)
	LYS716	4.24897	Electrostatic (Pi–Cation)
	PHE712	3.80469	Other (Pi–Sulfur)
	TYR32	5.21594	HYD (Pi–Pi T‐shaped)
	VAL36	5.13086	HYD (Pi–Alkyl)
	VAL744	4.81606	HYD (Pi–Alkyl)
	LYS716	3.84364	HYD (Pi–Alkyl)
	MET746	5.19942	HYD (Pi–Alkyl)
**7**	GLU45	2.76733	HB; Electrostatic (Salt bridge; attractive charge)
	GLU45	3.01842	Electrostatic (Attractive charge)
	GLU45	2.31139	HB (Conventional)
	MET44	5.01223	Other (Pi–Sulfur)
	LYS10	4.47408	HYD (Alkyl)
	LYS10	4.08024	HYD (Alkyl)
	LEU11	4.64777	HYD (Pi–Alkyl)
	LYS10	5.3622	HYD (Pi–Alkyl)

**Abbreviations**: HB, hydrogen bond; HYD, hydrophobic interaction.

**TABLE 10:** XO‐compounds **1–7** interaction categories, species, and molecular docking distance.

Compound number	Interacting amino acids	Interaction distance (Å)	Interaction types
**1**	LYS754	2.36984	HB (Conventional)
	PRO753	2.76781	HB (Conventional)
	GLU1065	3.63982	HB (Carbon)
	LYS754	3.96741	Electrostatic (Pi–Cation)
	LYS754	2.57993	HYD (Pi–Sigma)
	LYS792	3.62306	HYD (Alkyl)
	ILE596	4.84481	HYD (Alkyl)
	LYS792	4.41113	HYD (Alkyl)
	PHE763	4.89009	HYD (Pi–Alkyl)
	VAL591	4.31358	HYD (Pi–Alkyl)
	ILE596	4.50568	HYD (Pi–Alkyl)
**2**	GLU402	2.48847	HB (Conventional)
	LEU398	2.21508	HB (Conventional)
	GLY399	2.57793	HB (Conventional)
	ILE353	2.77834	HYD (Pi–Sigma)
	ALA301	4.10546	HYD (Alkyl)
	LYS395	4.78107	HYD (Alkyl)
	ILE353	4.80384	HYD (Alkyl)
	ILE353	4.64956	HYD (Alkyl)
	LEU404	4.83326	HYD (Alkyl)
	LEU257	4.39599	HYD (Pi–Alkyl)
**3**	ARG37	2.11079	HB (Conventional)
	LYS754	2.58567	HB (Conventional)
	GLY35	3.5869	HB (Carbon)
	PRO753	3.53071	HB (Carbon)
	ARG37	2.5811	HYD (Pi–Sigma)
	LEU36	5.09137	HYD (Alkyl)
	ILE596	4.14076	HYD (Alkyl)
	ILE596	4.35261	HYD (Alkyl)
	PHE763	4.57591	HYD (Pi–Alkyl)
	PHE763	4.15478	HYD (Pi–Alkyl)
	VAL591	5.24646	HYD (Pi–Alkyl)
	LYS754	4.29431	HYD (Pi–Alkyl)
	VAL591	4.94411	HYD (Pi–Alkyl)
	ILE596	4.44077	HYD (Pi–Alkyl)
	PRO597	4.86075	HYD (Pi–Alkyl)
**4**	GLU402	1.89505	HB (Conventional)
	ALA301	3.71237	HB (Carbon)
	LYS256	2.76628	HB; electrostatic (Pi–Cation; pi–donor HB)
	LEU257	2.7108	HYD (Pi–Sigma)
	ALA301	3.66446	HYD (Alkyl)
	LEU257	5.28896	HYD (Alkyl)
	VAL259	4.7531	HYD (Alkyl)
	ILE353	4.05197	HYD (Pi–Alkyl)
**5**	THR1077	2.28954	HB (Conventional)
	GLN1040	3.55817	HB (Carbon)
	ARG912	4.71592	Electrostatic (Pi–Cation)
	GLN1040	2.67162	HYD (Pi–Sigma)
	PHE798	5.26992	HYD (Pi–Pi stacked)
	SER1080	4.54352	HYD (Amide–Pi stacked)
	LYS1045	4.81965	HYD (Alkyl)
	VAL1259	5.04819	HYD (Alkyl)
	ARG912	5.14502	HYD (Pi–Alkyl)
	MET1038	4.98097	HYD (Pi–Alkyl)
**6**	THR354	2.86522	HB (Conventional)
	LEU257	2.21434	HB (Conventional)
	LEU257	2.98217	HB (Conventional)
	PRO253	3.52947	HB (Carbon)
	LYS256	2.56221	HB; electrostatic (Pi–Cation; pi–donor HB)
	ALA301	3.68991	HYD (Alkyl)
	VAL259	3.94041	HYD (Alkyl)
	ILE353	4.86268	HYD (Alkyl)
	LEU404	4.35041	HYD (Alkyl)
	LEU257	4.73088	HYD (Pi–Alkyl)
	ILE353	4.82785	HYD (Pi–Alkyl)
	LYS256	5.14562	HYD (Pi–Alkyl)
**7**	GLU1261	5.50442	Electrostatic (Attractive charge)
	GLU1261	5.28181	Electrostatic (Attractive charge)
	THR1077	2.31011	HB (Conventional)
	GLN1040	3.6092	HB (Conventional)
	SER1080	3.75724	HB (Carbon)
	ARG912	4.45056	Electrostatic (Pi–Cation)
	GLN1040	2.79314	HYD (Pi–Sigma)
	SER1080	2.85748	HYD (Pi–Sigma)
	PHE798	5.56161	HYD (Pi–Pi stacked)
	SER1080	4.44401	HYD (Amide–Pi stacked)
	LYS1045	5.34704	HYD (Alkyl)
	VAL1259	4.91348	HYD (Alkyl)
	ARG912	4.82947	HYD (Pi–Alkyl)
	MET1038	5.35573	HYD (Pi–Alkyl)

**Abbreviations**: HB, hydrogen bond; HYD, hydrophobic interaction.

To examine the consistency between the computational predictions and the experimental enzyme inhibition results, the docking scores were compared with the experimentally determined IC_50_ values of the seven compounds (Table 7). Since lower IC_50_ values indicate stronger experimental inhibition, whereas more negative docking scores represent more favorable predicted ligand–enzyme interactions, Spearman's rank correlation analysis was used to evaluate the relationship between the two parameters. For urease, a moderate‐to‐strong correlation was observed between docking score and experimental IC_50_ (*ρ* = 0.667, *p* = 0.102). Although this relationship did not reach statistical significance, the overall ranking showed partial consistency between the computational and experimental results. In contrast, the XO data exhibited a very strong and statistically significant correlation (*ρ* = 0.929, *p* = 0.0025), indicating a high degree of agreement between the docking‐based ranking and the experimentally determined inhibitory potency.

The comparison also illustrates that docking predictions do not reproduce experimental inhibition uniformly across the two enzymes. For urease, for example, compound **6** showed the strongest experimental inhibition (IC_50_ = 8.67 µg/mL), whereas compound **1** exhibited a slightly more favorable docking score (−7.50 kcal/mol compared with −7.30 kcal/mol for compound **6**). This difference illustrates that docking scores should not be interpreted as direct predictors of IC_50_ values. For XO, however, the compounds with the most favorable docking scores, particularly compounds **3**, **4,** and **7**, were also among those showing the strongest experimental inhibition, which is consistent with the significant correlation observed.

The interaction analysis further indicated that the compounds established hydrogen‐bonding, electrostatic, and hydrophobic contacts with residues located within or around the enzyme binding regions (Tables 9 and 10). These interactions provide a structural basis for understanding how the compounds may be accommodated within the catalytic sites. Nevertheless, the presence of a particular interaction or a more favorable docking score does not by itself establish the precise inhibition mechanism.

Overall, the correlation analysis provides quantitative evidence that the docking predictions, particularly for XO, are consistent with the experimental enzyme inhibition results. However, the docking calculations remain complementary to the experimental assays and should not be considered definitive measurements of docking score or inhibition mechanism. Differences between computational and experimental results may reflect factors not fully represented in conventional docking calculations, including protein flexibility, solvent effects, entropic contributions, induced‐fit effects, protonation states, and enzyme kinetic behavior. Further molecular dynamics and binding free‐energy calculations would be valuable for evaluating the temporal stability and energetic characteristics of the predicted enzyme–ligand complexes in future studies.

## 3 Conclusions

A new series of thiosemicarbazones bearing Schiff base (**1**–**7**) was effectively synthesized from 4‐(dimethylamino)benzaldehyde and thiosemicarbazides. The compounds were obtained in moderate to good yields with 63%–84%. Their structures were confirmed by IR, ^1^H, and ^13^C NMR spectroscopic techniques, as well as elemental analysis. The synthesized derivatives exhibited IC_50_ values ranging from 8.50 ± 0.14 to 31.44 ± 1.39 µg/mL, indicating varying degrees of antioxidant potency. The newly synthesized compounds, except for compound **6** (IC_50_: 8.50 ± 0.14 µg/mL), showed higher activity as a radical scavenger (DPPH^•^) than the standard Trolox (25.90 ± 0.42 µg/mL). Compound **6** displayed the strongest antioxidant activity among the synthesized compounds. The observed differences in antioxidant activity suggest that structural modifications may influence the radical‐scavenging properties of thiosemicarbazone derivatives. The nature and position of the substituent groups also affected urease inhibition, with compound **6** being the most effective. Compounds **1** and **2** followed closely behind, outperforming the standard thiourea, while the other products showed lower activity than the standard. The compounds with electron‐withdrawing groups (**3**–**5**) showed more potent XO inhibition than allopurinol, while electron‐donating groups in compounds 7, 1, and 2 were associated with weaker activity. The molecular docking analysis provided complementary structural insights into the possible interactions of the active compounds with the enzyme binding sites. I offered a plausible structural rationale for the experimentally observed inhibition. Nevertheless, the docking results should not be regarded as direct quantitative measures of inhibitory potency or definitive evidence of the underlying inhibition mechanism.

### 3.1 Limitation

A limitation of the present study is that chromatographic purity was not assessed by HPLC. The compounds were characterized using the available spectroscopic and analytical data, including elemental analysis; therefore, the absence of HPLC‐based purity data should be considered when interpreting the characterization results.

## 4 Experimental Section

### 4.1 Materials

Chemical reagents and solvents (spectroscopic grade) were sourced from suppliers such as Thermo Fisher, Sigma–Aldrich, and Merck and used as received without further treatment. A Eurovector EA3000‐Single analyzer was employed for the elemental analysis. The melting points of the synthesized derivatives were measured using a Stuart SMP30 device and are presented without further correction. For spectroscopic characterization, FT‐IR spectra were acquired using a Bruker Alpha spectrometer. In contrast, ^1^H and ^13^C NMR spectra were recorded on a Bruker Avance DPX spectrometer at 400 MHz, using DMSO‐*d_6_* as the NMR solvent. Electronic absorption spectra (UV–vis) were obtained using a Shimadzu Pharmaspec 1700 spectrophotometer. The following abbreviations describe the NMR splitting patterns: s (singlet), d (doublet), dd (doublet of doublets), t (triplet), and m (multiplet).

### 4.2 General Procedure for Synthesis of Thiosemicarbazones Bearing Schiff Base (**1–7**)

To synthesize the thiosemicarbazide intermediates, hydrazine monohydrate (7.50 mmol) was reacted with equivalent amounts of different isothiocyanates in 20 mL of ethanol through dropwise addition under constant agitation, while maintaining the temperature in an ice bath. Following the reaction, the mixture was refrigerated overnight to facilitate precipitation. The crude thiosemicarbazide intermediates were then isolated through filtration, dried, and further purified with ethanol. To synthesize the final derivatives, the obtained thiosemicarbazides (4.00 mmol) were reacted with 4‐(dimethylamino)benzaldehyde (4.00 mmol) in 20 mL of aqueous ethanol, catalyzed by a few drops of hydrochloric acid. The solution was stirred under reflux at 78°C for 3–5 h. The resulting solids were filtered, washed, and dried under ambient conditions. As depicted in Scheme 1, the target Schiff base‐thiosemicarbazone hybrids were obtained in favorable yields ranging from 63% to 84%, following a slightly modified version of the literature protocols [33, 50].

**SCHEME 1:**
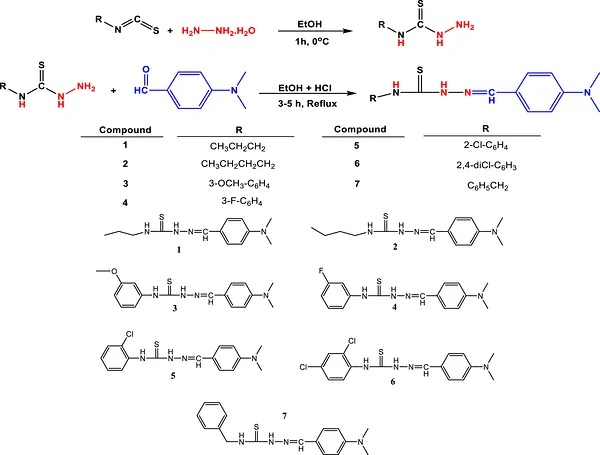
Synthesis procedure and chemical structures of new thiosemicarbazones (**1**–**7**).

### 4.3 Antioxidant Activity Assay

The DPPH^•^ (1,1‐diphenyl‐2‐picryl‐hydrazil) assay was employed to evaluate the free radical scavenging potential of the tested compounds [51, 52]. To evaluate antioxidant capacity, 150 µL of various concentrations of the synthesized compounds or Trolox (standard) were combined with 50 µL of a 0.1 mM DPPH^•^ solution in a 96‐well plate. Following a 30‐min incubation period in a dark environment at ambient temperature, the absorbance of each well was recorded at 517 nm utilizing a BIOTEK (Epoch2) microplate reader. The inhibitory concentrations (IC_50_) were subsequently calculated and expressed in µg/mL [53].

### 4.4 XO Inhibition Activity Assay

To evaluate the inhibitory effects, 50 µL of the samples (or allopurinol as a reference) were mixed with 100 µL of 0.041 mM xanthine in a 96‐well microplate. Subsequently, 50 µL of XO (0.1 U/mL in pH 7.5 phosphate buffer) was introduced to initiate the reaction. After a 5‐min incubation period at 37°C, 100 µL of 1 M HCl was added to terminate the enzymatic activity. The final absorbance of the mixture was recorded at 292 nm using a UV/vis spectrophotometer [54]. IC_50_ (µg/mL) values were calculated using the absorbance values.

### 4.5 Urease Inhibition Activity Assay

The indophenol technique was used to measure the materials' urease inhibitory activity [55]. The urease inhibitory potential was evaluated by combining 50 µL of 17 mM urea, 25 µL of 1 U urease (prepared in 100 mM Na–K buffer at pH 8.2), and 10 µL of the synthesized derivatives or standard thiourea in a 96‐well plate. Following a 15‐min incubation period at 30°C, the reaction was supplemented with 45 µL of phenol reagent (containing 8% phenol and 0.1% sodium nitroprusside) and 70 µL of alkaline reagent (comprising 2.5% NaOH and 4.7% NaOCl). The resulting mixture was kept at 30°C for an additional 50 min. Absorbance measurements were performed at 630 nm utilizing a BIOTEK (Epoch2) microplate reader, and the inhibitory efficiency was expressed as IC_50_ values (µg/mL).

### 4.6 Molecular Docking Study

Molecular docking simulations are a powerful computational tool for identifying ligands that exhibit optimal geometric and energetic complementarity to the active sites of target proteins. This approach is also instrumental in characterizing the specific non‐covalent interactions and docking scores established between the ligands and their biological targets [56]. The molecular docking simulations in the present study were performed utilizing the AutoDock Vina software package (version 4.2) [57, 58]. The docking orientations with the most favorable binding energies were examined and visualized using the Discovery Studio Visualizer 2021 software package.

#### 4.6.1 Protein Preparation

Computational docking simulations were conducted on the urease and XO catalytic domains, recognized as primary targets for stomach disease in drug discovery. The 3D crystallographic coordinates of urease and XO, characterized by X‐ray diffraction at a 1.49 and 1.80 Å resolution, were retrieved from the RSCB Protein Data Bank using PDB IDs 4GY7 and 3NRZ, respectively. Structural refinements of the proteins were performed using BIOVIA Discovery Studio, which included the addition of missing hydrogen atoms and terminal residues. Following energy minimization, water molecules not involved in co‐crystallization or ligand‐binding interactions were removed. The structural integrity and final 3D conformations of the target proteins were validated using Swiss PDB Viewer 4.10.

#### 4.6.2 Protein–Ligand Docking

The protein–ligand docking simulations were executed using the AutoDock Vina (version 4.2) software interface. During the computational analysis, the target protein structures were treated as rigid bodies, whereas the ligand molecules were permitted full conformational flexibility to ensure optimal binding pose identification [59]. The Open Babel module was utilized to convert and store both protein and ligand structures in the ‘.pdbqt’ file format. A grid box was established to encompass the active binding site, with its dimensions and center coordinates precisely adjusted based on the site's boundaries. In this study, a semi‐flexible docking approach was implemented using the Lamarckian genetic algorithm (LGA) integrated within AutoDock Vina. Following the simulations, the software generated docking scores in ‘.txt’ format, categorized into various conformers. To evaluate the specific non‐covalent interactions between the ligands and the active site residues, the resulting output files were analyzed using Discovery Studio Visualizer 2021. The most favorable binding pose was identified by considering the highest docking score and the most robust intermolecular interaction profile.

### 4.7 Statistical Analysis

All biological assays were performed in triplicate, and the data are presented as mean ± standard deviation. Statistical evaluations were conducted using SPSS 20.0 software. For data sets exhibiting a normal distribution, the homogeneity of means across multiple groups was assessed. Tukey's HSD test was employed for post hoc multiple comparisons to identify significant differences. Statistical significance was defined as *p* < 0.05, indicating meaningful variations between the test compounds, standards, and the respective activity values.

To quantitatively assess the relationship between computational docking predictions and experimental enzyme inhibition, Spearman rank correlation analysis was performed between the docking scores and the experimentally determined IC_50_ values for compounds **1**–**7** against urease and XO. Spearman's correlation was selected because docking scores and IC_50_ values represent different scales and the relationship was evaluated primarily according to the relative ranking of compounds. Lower IC_50_ values and more negative docking scores were considered indicative of stronger experimental inhibition and more favorable predicted interactions, respectively. A *p* value < 0.05 was considered statistically significant.

## Author Contributions

**Hasan Yakan**: Spectroscopic characterization, conceptualization, writing – original draft, writing – review, and visualization and editing. **Halil Muglu**: Synthesis, characterization, formal analysis, investigation, and writing – review. **Semiha Yenigun**: Biological studies, molecular docking, software, data curation, writing – original draft, and writing – review. **Tevfik Ozen**: Biological studies, molecular docking, software, data curation, and writing – review. All authors reviewed and approved the article.

## Funding

The authors have nothing to report.

## Ethics Statement

The authors have nothing to report.

## Conflicts of Interest

The authors declare no conflicts of interest.

## Supporting information




**Supporting File 1**: cbdv71882‐sup‐0001‐SuppMat.docx.

## Data Availability

All spectra of the compounds are given in the Supporting Information.
