# Supplementary material for: Preparation, Spectroscopic Elucidation, Antioxidant Potential, Enzyme Inhibition, and Molecular Docking Studies of Novel Schiff Base‐Derived Thiosemicarbazones
Source: Chem Biodivers. 2026 Oct 30;23(11):e71882. doi: 10.1002/cbdv.71882 (PMC13720966; doi:10.1002/cbdv.71882)
Supplement: Supplementary file 1 [file CBDV-23-e71882-s001.docx]

**SUPPLEMENTARY MATERIAL**

**Preparation, Spectroscopic Elucidation, Antioxidant Potential, Enzyme Inhibition, and Molecular Docking Studies of Novel Schiff Base-Derived Thiosemicarbazones**

**Hasan Yakan^1^, Halit Muğlu^2^, Semiha Yenigun^3^*, Tevfik Ozen^3^***

**^1^** Ondokuz Mayis University, Faculty of Education, Department of Chemistry Education, 55139, Atakum, Samsun, Türkiye.

**^2^** Kastamonu University, Faculty of Science, Department of Chemistry, 37150, Kastamonu, Türkiye.

**^3^** Ondokuz Mayis University, Faculty of Sciences, Department of Chemistry, 55139, Atakum, Samsun, Türkiye.

Figure S1. IR spectrum of compound **1**

Figure S2. IR spectrum of compound **2**

Figure S3. IR spectrum of compound **3**

Figure S4. IR spectrum of compound **4**

Figure S5. IR spectrum of compound **5**

Figure S6. IR spectrum of compound **6**

Figure S7. IR spectrum of compound **7**

Figure S8. ^1^H NMR spectrum of compound **1**

Figure S9. ^1^H NMR spectrum of compound **2**

Figure S10. ^1^H NMR spectrum of compound **3**

Figure S11. ^1^H NMR spectrum of compound **4**

Figure S12. ^1^H NMR spectrum of compound **5**

Figure S13. ^1^H NMR spectrum of compound **6**

Figure S14. ^1^H NMR spectrum of compound **7**

Figure S15. ^13^C NMR spectrum of compound **1**

Figure S16. ^13^C NMR spectrum of compound **2**

Figure S17. ^13^C NMR spectrum of compound **3**

Figure S18. ^13^C NMR spectrum of compound **4**

Figure S19. ^13^C NMR spectrum of compound **5**

Figure S20. ^13^C NMR spectrum of compound **6**

Figure S21. ^13^C NMR spectrum of compound **7**
